# HPV-Induced Anal and Peri-Anal Neoplasia, a Surgeon’s Experience: 5-Year Case Series

**DOI:** 10.3390/diagnostics13040702

**Published:** 2023-02-13

**Authors:** Christoforos Kosmidis, Christina Sevva, Vasiliki Magra, Nikolaos Varsamis, Charilaos Koulouris, Ioannis Charalampous, Konstantinos Papadopoulos, Panagiota Roulia, Marios Dagher, Vasiliki Theodorou, Chrysi Maria Mystakidou, Isaak Kesisoglou

**Affiliations:** 1 3rd Surgical Department, University General Hospital of Thessaloniki “AHEPA”, School of Medicine, Faculty of Health Sciences, Aristotle University of Thessaloniki, 1st St. Kiriakidi Street, 54621 Thessaloniki, Greece; 2 European Interbalkan Medical Center, 10 Asklipiou Street, 55535 Pylaia, Greece; 3 Medical School, Faculty of Health Sciences, Aristotle University of Thessaloniki, 54124 Thessaloniki, Greece

**Keywords:** condylomata acuminata, HPV, abdominoperineal resection, vaccination

## Abstract

*Purpose*: One of the most known sexually transmitted diseases is Condylomata acuminata (CA), a skin lesion occurring due to infection from Human Papilloma Virus (HPV). CA has a typical appearance of raised, skin-colored papules ranging in size from 1 mm to 5 mm. These lesions often form cauliflower-like plaques. Depending on the involved HPV-subtype (either high-risk or low-risk) and its malignant potential, these lesions are likely to lead to malignant transformation when specific HPV subtypes and other risk factors are present. Therefore, high clinical suspicion is required when examining the anal and perianal area. *Methods*: In this article, the authors aim to present the results of a five-year case series (2016–2021) of anal and perianal cases of CA. *Results*: A total of 35 patients were included in this study. Patients were categorized based on specific criteria, which included gender, sex preferences, and human immunodeficiency virus infection. All patients underwent proctoscopy and excision biopsies were obtained. Based on dysplasia grade patients were further categorized. The group of patients where high-dysplasia squamous cell carcinoma was present was initially treated with chemoradiotherapy. Abdominoperineal resection was necessary in five cases after local recurrence. *Conclusions*: CA remains a serious condition where several treatment options are available if detected early. Delay in diagnosis can lead to malignant transformation, often leaving abdominoperineal resection as the only option. Vaccination against HPV poses a key role in eliminating the transmission of the virus, and thus the prevalence of CA.

## 1. Introduction

Condylomata acuminata (CA), otherwise known as anogenital warts, are caused by human papilloma virus (HPV) infection. HPV is one of the commonest sexually transmitted diseases worldwide, with 9–13% of the world population affected [1,2], commonly between 20 and 39 years old [1].

HPV is a non-enveloped, double-stranded DNA virus from the papilloma virus family that infects the nucleus of differentiated squamous cells [1,3]. It can remain in a latent state, with an incubation period from one month to two years after infection [1]. More than 200 [3] subtypes have been described, which are subdivided into high-risk (HR-HPV) and low-risk types (LR-HPV) based on their oncogenic potential [4]. More than 40 types are known to affect the anogenital area [1]. Low-risk types 6 and 11 are responsible for over 90% of condylomata acuminata [4]. High-risk types 16 and 18 have been solidly linked to cervical, anal, and oral malignancy [4]. HPV infection appears to be the cause for most anal cancers and virtually all cases of cervical cancer [5]. Due to its structure, the viral DNA integrates into the host human genome causing malignant transformation and eventually evolving to cancer. Evolution to cancer occurs mostly through actions affecting two major paths: alterations in cell cycle regulation and telomeres combined with blockage in apoptosis. These interventions cause DNA instability, leading eventually to genomic damage, and together with the disabled tumor suppressor paths, mostly protein p53, result in the progression to malignancies [6,7].

The HPV genome consists of a transcribed region which encodes six early proteins, namely E1, E2, E4, E5, E6, and E7. Some of these HPV proteins are strongly type-specific and they can be detected in certain neoplastic lesions. Especially in the HR-HPV subtypes increased levels of E5, E6, and E7 oncoproteins seem to affect and activate multiple signaling pathways and thus stimulate proliferation of infected cells. Although life-cycle organization between HR- and LR-HPV subtypes is similar, the main difference lies in the strongly different ability of each group’s oncoproteins (mostly E6 and E7) to control cell cycle entry in the basal/parabasal cell layers [8,9].

HPV invades the cells of the epidermal basal layer through microabrasions [3]. Transmission occurs via human-to-human contact, smear infection, or vertically [4]. Several types of HPV exist which show a different biological behavior. Based on this behavior, the HPV subtypes are further categorized into low-risk and high-risk types according to their malignant potential. Low-risk types (also known as non-oncogenic types) include HPV 6, 11, 40, 42, 43, 44, 54, 61, 70, 72, 81, and CP6108 and are mainly responsible for causing anogenital warts, low-grade changes in cells of the cervix and recurrent respiratory papillomatosis [6,10]. The oncogenic high-risk HPV types include HPV 16, 18, 31, 33, 35, 39, 45, 51, 52, 56, 58, 59, 66, 68, 73, and 82. HPV 16 and 18 seem to have the higher malignant potential as they are linked with high-grade dysplasia and invasive carcinoma of the cervix and the anus in both men and women [6,11].

High-risk HPV subtypes are responsible for certain types of cancer of the cervix (cervical intraepithelial neoplasia (CIN)), anus, penis, vagina, vulva, oropharynx and even esophageal adenocarcinoma (EAC). Subtypes HPV-16 and HPV-18 are held responsible for the majority of cervical cancers and almost 95% of HPV-positive oropharyngeal cancers (OPCs). Other types of high-risk HPV related neoplasia are the vulvar high-grade squamous intraepithelial lesions (vH-SIL) which can progress to invasive vulvar cancer. From the low-risk HPV subtypes, some persistent ones, such as HPV-6 and HPV-11, cause recurrent conditions and mostly involve anogenital warts, respiratory papillomatosis, and vulvar low-grade squamous intraepithelial lesions (vL-SIL), which are considered to represent a benign condition [12,13].

Risk factors for infection include early-onset sexual contact, multiple sexual partners, high-risk sexual practices, concomitant infection with other sexually transmitted diseases (HIV, chlamydia, gonorrhea, HBV), and poor hygiene [4]. Regular use of condoms can protect against HPV infection but cannot fully prevent it. HPV vaccination prior to first sexual contact prevents infection with the subtypes contained in the vaccine. Available vaccines are the 9-valent for types 6, 11, 16, 18, 31, 33, 45, 52, 58 (Gardasil^®^9), the quadrivalent vaccine for types 6, 11, 16, and 18 (Gardasil^®^), and the bivalent vaccine for the high-risk types 16 and 18 (Cervarix^®^). In Greece, HPV vaccination is offered to girls 11–18 years old and boys and girls 11–26 with risk factors (HIV infection, transplant patients, history of malignancy, autoimmune disease, and immunocompromised patients) [14].

**Figure 1:**
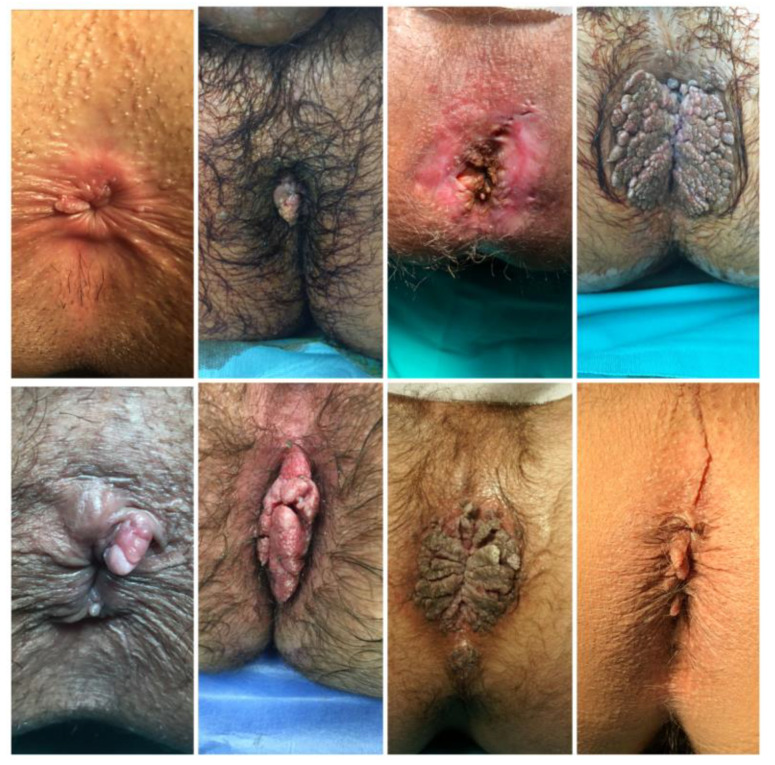
Different typical clinical presentation of CA as single papules or cauliflower-like plaques.

**Figure 2:**
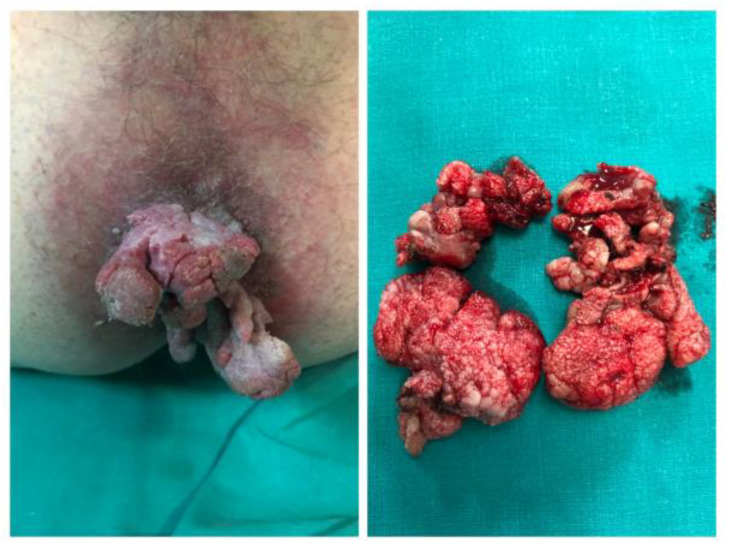
Clinical presentation of Giant CA (Bushke-Lowenstein tumor) and image of the specimen after excision.

HPV contains an oncogene that triggers cell proliferation using the three major viral oncoproteins mentioned above (E5, E6, E7) [6]. As the number of infected cells increases, epidermal layers thicken, leading to the macroscopic appearance of condylomata acuminata [1]. CA are typically diagnosed clinically due to their characteristic appearance. They are raised, skin-coloured papules ranging in size from 1 mm to 5 mm. These can coalesce, forming cauliflower-like plaques. CA can be flat or pedunculated lesions (Figure 1). In rare cases, they can form giant lesions, taking up the entire anogenital region. In such cases, Buschke-Lowenstein tumors or Giant Condylomata Acuminata caused by HPV infection should be considered. Bushke-Lowenstein tumor is categorized by some authors as a rare form of highly differentiated squamous cell carcinoma (SCC), although a globally accepted definition is not available in the literature [15,16] (Figure 2). Assessment of anal and peri-anal lesions is not complete without proctoscopy and in some cases sigmoidoscopy in order to identify lesions in the anal canal. Regular follow-up should follow initial diagnosis and treatment as there is a 2–4% risk of malignant transformation.

Herein we present a 5-year case series (2016–2021) of anal and perianal CA.

## 2. Materials and Methods

The patients selected had surgical resection of anal and perianal warts between 1/1/2016 and 1/1/2021 (60 months) in the 3rd Surgical Department of University General Hospital of Thessaloniki AHEPA, by a single surgical team. Patient data were collected using the histopathological reports of the specimens resected, which were acquired from the Histopathology Laboratory of Aristotle University of Thessaloniki, along with the patient records from the 3rd Surgical Department’s archive. Inclusion criteria included excision of anal and perianal lesions and exclusion criteria included final histopathological results of CA with high grade, mild-moderate dysplasia, and SCC.

## 3. Results

**Figure 3:**
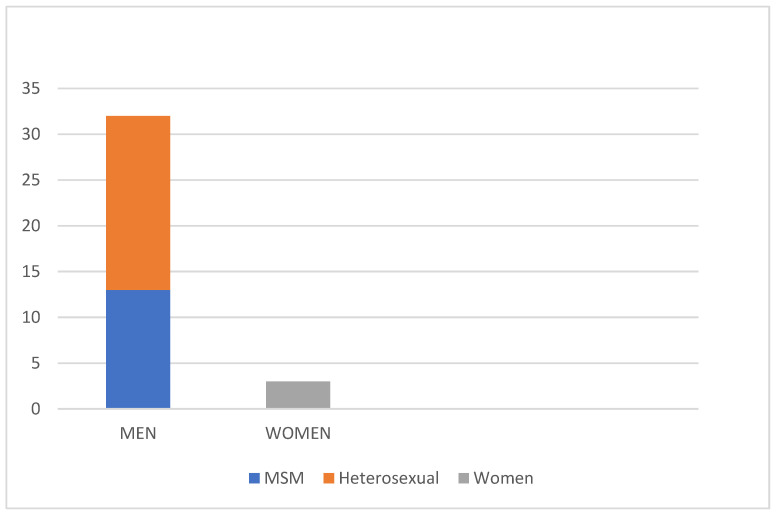
Distribution of the patients in our study based on sex. The vast majority being male (91.4%), 37% of which were MSM.

**Figure 4:**
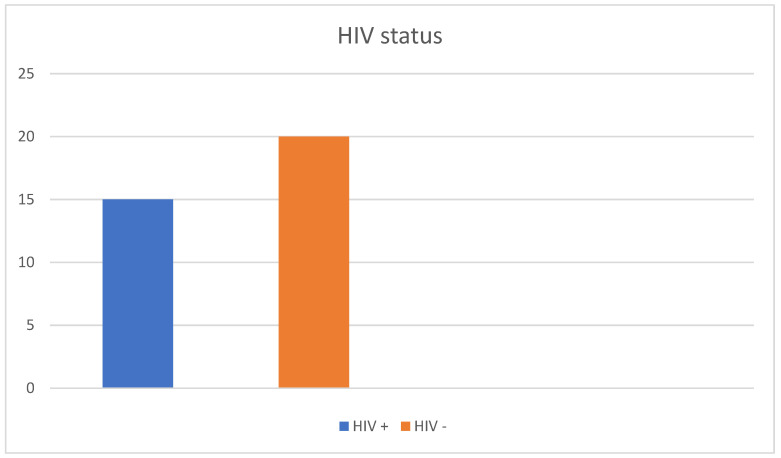
Prevalence of HIV in our patient cohort. Nearly half (42.8%) of the patients were HIV positive.

A total of 35 patients were included in this study. The patient cohort consisted mostly of men: 32 patients were male (91.4%) and three were female (8.6%), with an average age of 39.9 years. The patients were between the age of 17 and 73 years. Moreover, 13 of the male patients (37.1%) reported to have sex with men (MSM) (Figure 3 and Table 1. In addition, 15 patients were HIV positive (42.8%) (Figure 4 and Table 1). All HIV patients had CD4 levels in the normal range. None of the patients were immunized against HPV.

**Figure 5:**
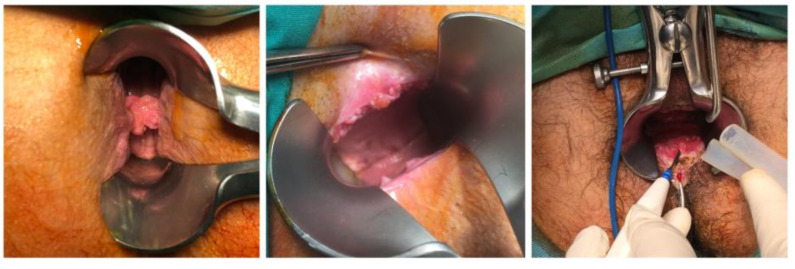
Lesions of CA revealed during proctoscopy using Eisenhammer Rectal Speculum.

All patients had proctoscopy under general anesthesia and excision biopsy of all suspicious perianal and anal lesions (Figure 5). Excision was performed using energy sources, specifically diathermy and radiofrequency-driven bipolar electrosurgical devices. All patients were administered topical agents, imiquimod or sinecatechins, post-operatively. All patients were followed up or remain subject to regular follow ups according to international guidelines for five years. Further, 16 patients required a second procedure to remove condylomata after relapse (45.7%) within this five-year follow up time period. All women treated were sent for a gynecology consult prior to excision biopsy and underwent a colposcopy and Pap smear test in order to rule out the presence of cervical intraepithelial neoplasia (CIN).

**Figure 6:**
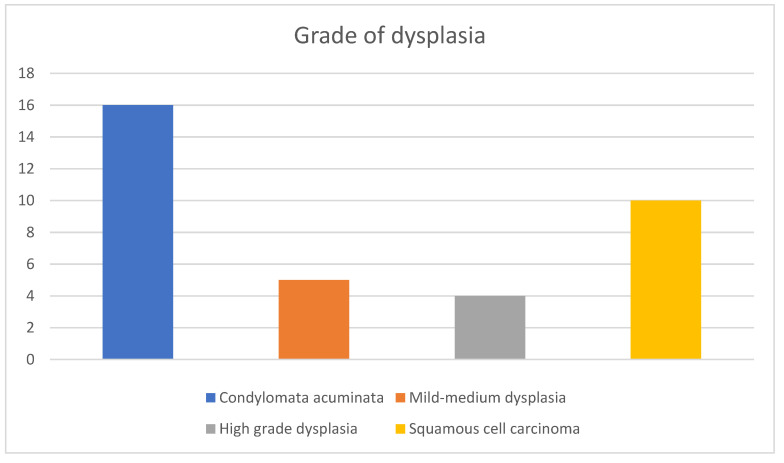
Grades of dysplasia in the histopathology results of the cohort, ranging from CA without dysplastic/neoplastic features to invasive SCC. A total of 19 patients (54.7%) had dysplastic and malignant features in their samples ranging from mild (5 patients) and high (4 patients) grade dysplasia to confirmed SCC (10 patients), illustrating the prevalence of malignant transformation in the context of CA and the importance of follow up.

**Figure 7:**
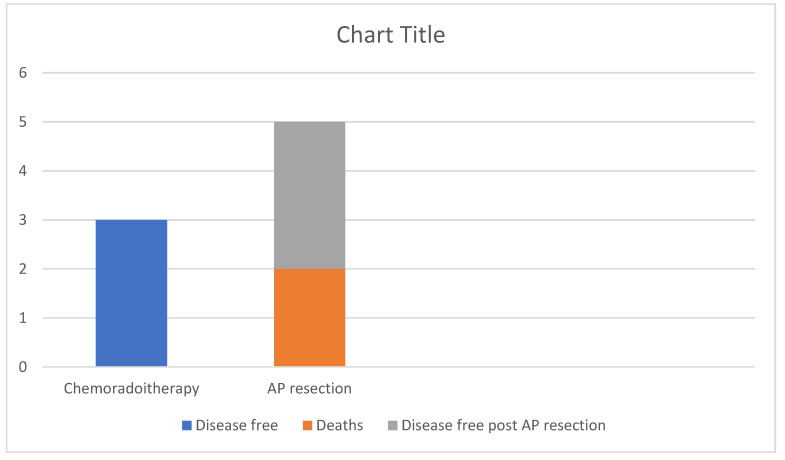
Outcomes of the patients with invasive SCC. 50% remain disease free after chemoradiotherapy alone, while of the 50% that required AP resection after relapse 60% remain disease free at 5 years post-operatively and 40% died.

Histopathology reports illustrate 16 patients with condylomata acuminata without dysplastic or neoplastic features (45.7%). Five patients had mid- to high grade dysplasia (14.3%); four patients had high grade dysplasia (11.4%); 10 patients (28.6%) were found to have invasive squamous cell carcinoma (SCC) in their histopathology results; two patients had Buschke-Lowenstein tumors (5.7%) (Figure 6 and Table 2. Interestingly enough, out of the 15 HIV (+) patients, only five appeared to have malignant lesions at the histopathological examination. However, not all of these malignant lesions showed a recurrent pattern in immunosuppressed patients. Four out of five patients that needed to undergo abdominoperineal resection appeared to be HIV (+). From the HIV (+) patients, five patients developed SCC, four patients showed some grade of dysplasia, and six patients developed no malignancies. Both the number of patients included and the number of immunosuppressed patients due to HIV infection are considered to be limited. Therefore, a safe correlation between HIV status and the occurrence of malignancy cannot be pointed out at this point. Patients with invasive SCC were initially treated with chemoradiotherapy, five (50%) of which required abdominoperineal resection after local recurrence. Three of the patients who underwent abdominoperineal resection remain disease free five years post-operatively, while the other two died in one and 3.5 years post-operatively, respectively (Figure 7 and Table 2).

The remaining 25 patients whose histopathology results showed no presence of confirmed malignancy did not receive any further treatment. Excision of the lesions was considered enough. These patients continue to have annual follow ups with proctoscopy and remain disease-free.

## 4. Discussion

Anal and perianal condylomata acuminata represent a controversial subject, largely due to limited clinical trials and high-level evidence, especially in terms of screening and prevention. There are currently no official guidelines regarding large scale vaccination for males and screening for anal SCC or HSIL (high grade squamous intraepithelial lesions). Although gender distribution appears to be roughly equal in the literature, the vast majority of the patients in this cohort were male (91.4%). This could be due to a number of factors. Nowadays, most women have regular screening for cervical cancer and consult their gynecologist frequently, who may manage a significant number of perianal warts. This department has a close cooperation with the HIV Medicine Unit of Aristotle University of Thessaloniki, which could account for the increased male predominance as well as the high percentage of HIV positive patients in this cohort.

Another significant observation to note is the large percentage of HSIL and invasive SCC. Interestingly, only a 2–4% rate of malignant transformation is reported in the literature, whereas we found 28.6% of our patient cohort had invasive SCC and nine patients (25.7%) had mid- to high grade dysplasia. HIV infection could, of course, contribute to the notably high percentage of dysplasia and invasive SCC, as it has been shown to be a risk factor for malignant transformation of CA [17,18,19]. There are currently no national standards for screening for HSIL or anal cancer in any country and insufficient evidence to support it from current literature [20,21,22]. However, recommendations for screening HIV positive patients with annual digital rectal examination, anoscopy, and/or anal Papanicolaou tests have been published by several organizations specializing in sexually transmitted infections (STIs) and colorectal diseases [20]. Clinical guidelines in regard to screening are expected to become more specific once the Anal Cancer HSIL (High-Grade Squamous Intraepithelial Lesions) Outcomes Research (ANCHOR) study is published [19,23,24]. The study started as a randomized clinical trial, which has been halted due to the therapy’s high success rates. It is a large phase 3 study awaiting publication.

Patient information and prevention remains poor. Current HPV vaccination guidelines in Greece include girls from 11 to 18 years old as well as boys and girls from 11 to 26 years old in high-risk groups (patients with autoimmune diseases, on immunosuppressant treatment, HIV positive, patients with malignancy and transplant patients) [10,23]. The mean age of our cohort (39.9 years) is higher than mean age of infection, but it remains striking that none of the patients were vaccinated, especially as the youngest was 17 years old. This reflects the level of information and possibly the lack of availability of the vaccine to a wider audience.

The biggest limitation of our study is the small sample size. However, the high percentage of HIV positive patients and the significant percentage of HSIL and SCC in our sample highlights the importance of regular follow up, as well as screening, especially in high-risk patients. It also illustrates the gap in preventive strategies and immunization in the male population. The need for further studies and high-level evidence in the management of CA and especially in the prevention of malignant transformation is indisputable.

Another important element regarding management of CA is high clinical suspicion. Physicians must be aware of the prevalence of this condition, the age-groups and other population sub-groups that are mostly affected, and its possible clinical manifestations. This verrucous hyperplasia can often be misdiagnosed due to its clinical presentation as multiple growths on the skin or mucous membranes [25]. They can often present also as papular to cauliflower-like lesions or as flat papular growths or as exophytic fronds [25]. Several other conditions, such as malignant lesions of the vulva, Paget’s disease of the anogenital region, molluscum contagiosum, psoriasis, acrochordon, sebaceous cysts, Buschke-Lowenstein tumors, secondary syphilis, seborrheic dermatosis, popular lichen planus, and even hemorrhoids, can make the differential diagnosis challenging [1,25,26].

Final diagnosis, however, is set only after excision of the lesions through biopsy and histopathological examination. Serology tools and methodologies, including polymerase chain reaction (PCR) detection and DNA detection assays, are not considered to be reliable with an estimated level of evidence of 2b. Moreover, it is not possible to have cultures of the virus. While the sample that can be used to detect the virus using PCR can be the same as the one used for cytological examination, several limitations are present regarding these methods. Most of the time, only a small sample is obtained, which can lead to sampling errors even when very sensitive assays are used. Another limitation concerns the menstrual cycle, which can also tamper with serology and molecular results [27,28]. Molecular diagnostic techniques for detection of HPV, although available, are not used as a standard screening procedure as it is believed that they would overestimate the proportion of women who have low-grade cytological abnormalities and do not also meet the criteria for a secondary prevention method (screening) [28,29]. Limitations also include the fact that various HPV genotypes can be present in each patient, while HPV persistence has been also identified as a key factor as a specific subtype cannot always be easy to identify. Therefore, the clinical utility of these serology and molecular HPV diagnostic tools requires careful management and evaluation. Overall, the adequate classification of patients into high- and low-risk groups requires accurate HPV mapping, underlying the need for further research in the domain of routine HPV molecular and serology detection [28].

Most HPV infections are subclinical and are usually handled and resolved by the immune system. HPV types can be further subcategorized to mucosal and cutaneous types. Mucosal types affect mucous membranes causing anogenital warts and neoplasia of the cervix. The squamous epithelium of the skin is infected by the cutaneous types, causing all forms of common warts, which can lead to CA. When specific oncogenic types of HPV are involved, the infected patient may develop cervical, oropharyngeal, anal, vulvar, vaginal cancer, and cancer of the penis [6,7,30].

While malignant potential and evolution to cancer strongly depends on the HPV type there are other risk factors that make certain patients more prone to an invasive type of cancer. These co-factors include mainly smoking, which seems to increase the risk of progression to high-grade dysplasia of the existing lesions, and presence of HIV infection. Immunosuppression caused by HIV significantly affects the behavior of HPV-related tumors as well as the outcome of those patients, findings that were confirmed also by the results of our cohort. The expected clearance of HPV from the immune system is significantly reduced in patients with HIV. Therefore HIV-positive patients develop HPV-related malignancies such as CA which tend to be more aggressive at a younger age and at an advanced stage upon diagnosis compared to HIV-negative patients [6]. Another important element regarding immunosuppressed individuals is that infection with LR-HPV subtypes can also lead to intraepithelial neoplasia through multiple infections which cannot be handled by the immune system [31]. In addition, in patients with genital warts and HIV infection, a greater resistance to standard treatment is observed [32].

**Figure 8:**
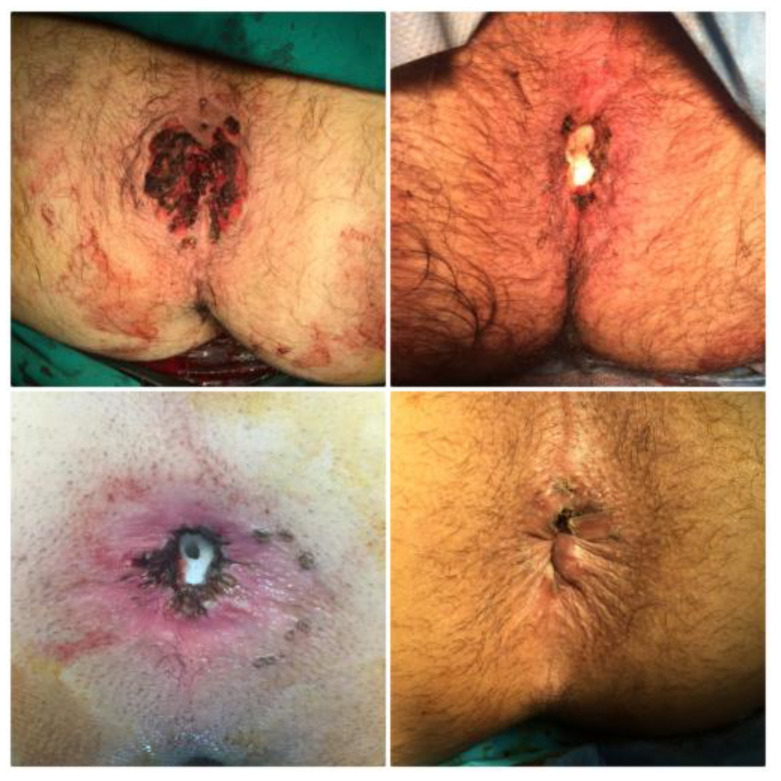
Final results after surgical excision of several CA lesions combined with electrocautery ablation, ultrasound scissors and diathermy.

**Figure 9:**
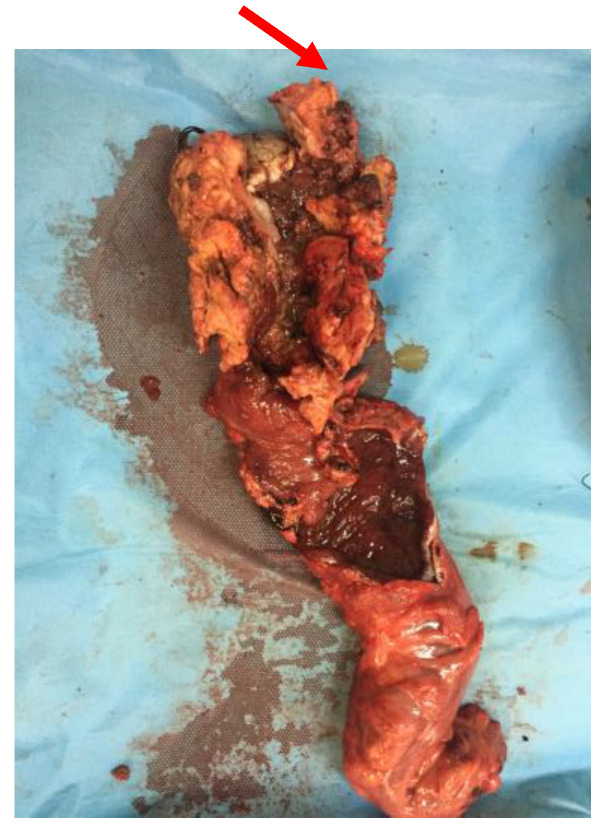
Specimen of abdominoperineal resection showing malignant transformation after local recurrence.

The optimal management of CA varies depending on the lesions’ size and location, but it usually includes surgical excision combined with another non-invasive technique (Figure 8). These techniques include electrocautery ablation, laser, photodynamic therapy, carbon dioxide (CO_2_), argon plasma coagulation, cryotherapy, local hyperthermia, and some medications [27,33,34,35]. Interestingly enough, medications that have been investigated for the treatment of CA include topical creams, such as 5-aminolevulinic acid (ALA), imiquimod 3.5%, and 5% trichloroacetic acid, podophyllotoxin, sinecatechins, and 5-fluorouracil [27,33,34,35,36,37]. Injection of Vitamin D3 has also been reported to achieve complete clearance of CA [38,39]. Several of these methods are used in combinations rather than as monotherapy. These not so commonly known techniques are usually being used as a curative alternative to conventional therapy methods in areas of the perineum where surgical excision is not a preferable option (e.g., penis, vulva) [33,36]. In cases of relapse, a surgeon might be left with the option of abdominoperineal resection, as presented in the results of our study (Figure 9).

While effective in removing the skin lesions, these treatment options do not offer a chance to eradicate HPV from the human body. While macroscopical lesions can be removed effectively and often without leaving any invaded margins, HPV cannot be cleared out from the human body. This leads to the realization that it is possible to have relapses and reoccurrence of HPV-related lesions whenever certain risk factors are present and/or the immune system appears more vulnerable. Important research has been conducted in order for immunotherapy to be established as a high-efficacy treatment option. Towards this direction, Mastutik et. al. studied protein p16INK4A expression, which seems to be highly related to HR-HPV infection and may serve as a biomarker for the prediction of malignancy potential in CA lesions [40].

While the eradication of HPV infection and HPV-related lesions especially in individuals with risk factors is far from available, the need for prevention must be underlined. Primary prevention through immunization is already applicable to young girls aged 10 years (e.g., Portugal) and older requiring two or three vaccination doses based on the age of first vaccination, although most national vaccination guidelines suggest a three-dose immunization scheme [29,41]. The real controversy is whether immunization should be made available to young males and male adults. A gender-neutral vaccination program was initially doubted by the vast majority of the global community as non-cost effective. Although the available data and sample of studies are limited, there is not yet a clear path towards the ideal strategy as nearly half of the available studies are in favor of expanding immunization to young boys. Nevertheless, it is advisable that countries make decisions after carrying out further studies based on the specific national epidemiological characteristics [42]. Secondary prevention poses also a very important role mostly in women, including screening with colposcopy and vaginal cytological smear examination. Examination of the perineum will reveal not only vaginal and cervical lesions, but also anal and perianal alterations of the squamous epithelium. Finally, tertiary prevention should not be discouraged: management of precancerous HPV-related lesions in early stages using surgical excision and biopsy provides individualized treatment and can positively affect the patient’s final outcome [29].

## 5. Conclusions

HPV remains one of the most frequently encountered STIs worldwide. Although important progress has been made in terms of preventing virus transmission through vaccine immunization, the virus remains untraceable in many cases. CA, as the clinical manifestation of HPV, affects a notable percentage of the population, especially young people. At this point, high clinical suspicion is required from all clinical doctors as CA has more treatment options and better prognosis if treated early. A delay in diagnosis can lead to malignant transformation, often leaving abdominoperineal resection as the only option. This study, although small in terms of the sample considered, aims to shed light on CA’s prevalence and patients’ special characteristics in a European country where not much data on the matter are available at the moment. Nevertheless, more studies are required in order to fully understand and target the most vulnerable population subgroups, raise awareness, and generalize the prevention of CA through immunization.

## Figures and Tables

**Table 1:** Distribution of patients based on sex, sexual preferences and presence of HIV infection.

	Male (n = 32)	Female (n = 3)	Total (n = 35)
MSM	13	0	13
HIV (+)	15	0	15

**Table 2:** Outcomes of patients of the study.

	No Dysplasia (n = 16)	Mild Dysplasia (n = 5)	High Grade Dysplasia (n = 4)	SCC (n = 10)	Total (n = 35)
Chemoradiotherapy	-			10	10
Relapse + Abdominoperineal resection	-	-	-	5	5
Disease free	16	5	4	8	33
Deaths	-	-	-	2	2

## Data Availability

Not applicable.
